# Altered Traffic of Cardiolipin during Apoptosis: Exposure on the Cell Surface as a Trigger for “Antiphospholipid Antibodies”

**DOI:** 10.1155/2015/847985

**Published:** 2015-09-29

**Authors:** Valeria Manganelli, Antonella Capozzi, Serena Recalchi, Michele Signore, Vincenzo Mattei, Tina Garofalo, Roberta Misasi, Mauro Degli Esposti, Maurizio Sorice

**Affiliations:** ^1^Department of Experimental Medicine, Sapienza University of Rome, Viale Regina Elena 324, 00161 Rome, Italy; ^2^Department of Hematology, Oncology and Molecular Medicine, National Institute of Health, Viale Regina Elena 299, 00161 Rome, Italy; ^3^Laboratory of Experimental Medicine and Environmental Pathology, Sabina Universitas, Via dell'Elettronica, 02100 Rieti, Italy; ^4^Italian Institute of Technology, Via Morego 30, 16136 Genoa, Italy

## Abstract

Apoptosis has been reported to induce changes in the remodelling of membrane lipids; after death receptor engagement, specific changes of lipid composition occur not only at the plasma membrane, but also in intracellular membranes. This paper focuses on one important aspect of apoptotic changes in cellular lipids, namely, the redistribution of the mitochondria-specific phospholipid, cardiolipin (CL). CL predominantly resides in the inner mitochondrial membrane, even if the rapid remodelling of its acyl chains and the subsequent degradation occur in other membrane organelles. After death receptor stimulation, CL appears to concentrate into mitochondrial “raft-like” microdomains at contact sites between inner and outer mitochondrial membranes, leading to local oligomerization of proapoptotic proteins, including Bid. Clustering of Bid in CL-enriched contacts sites is interconnected with pathways of CL remodelling that intersect membrane traffic routes dependent upon actin. In addition, CL association with cytoskeleton protein vimentin was observed. Such novel association also indicated that CL molecules may be expressed at the cell surface following apoptotic stimuli. This observation adds a novel implication of biomedical relevance. The association of CL with vimentin at the cell surface may represent a “new” target antigen in the context of the apoptotic origin of anti-vimentin/CL autoantibodies in Antiphospholipid Syndrome.

## 1. Changes in Phospholipid Distribution during Cell Apoptosis

Apoptosis or programmed cell death (PCD) constitutes a physiological phenomenon that concerns any nucleated cell but is particularly important in multicellular organisms, where it can be paradoxically considered a vital process. Apoptosis is critically important for fundamental processes, such as cell turnover, hormone-dependent atrophy, embryonic development, chemical-induced cell death, and immune system homeostasis [1–4].

Distinct morphological features and energy-dependent biochemical mechanisms characterize apoptosis versus other forms of cell death [2, 3]. In particular, apoptosis is accompanied by ultrastructural alterations, including cell shrinkage, cytoplasmic condensation, and DNA laddering [1, 4, 5], and by several biochemical modifications, such as protein cleavage, protein cross-linking, DNA breakdown, and phagocytic recognition [6]. Moreover, apoptosis has been reported to induce changes in the remodelling of membrane lipids (for a review, see [7]). Physiologically, eukaryotic cells maintain asymmetrical, organelle-specific distributions of membrane phospholipids. For example, phosphatidylcholine (PC) and sphingomyelin (SM) are almost exclusively located in the outer leaflet of the plasma membrane, while phosphatidylserine (PS) and 70% of phosphatidylethanolamine (PE) are located in the inner leaflet of the same membrane [8]. Many proapoptotic stimuli induce PS translocation to the outer membrane leaflet, which thus becomes a membrane “flag” on apoptotic cells and thereby acts as a recognition signal for phagocytosis [9–12].

Studies on transbilayer lipid movements during apoptosis have shown that PS translocation results from downregulation of the adenosine triphosphate-dependent aminophospholipid translocase and activation of a nonspecific lipid scramblase [13], both of which occur downstream caspase activation [6, 10]. Sorice et al. [14] reported lipid changes at the cell surface of lymphocytes that appeared to occur even before full caspase activation by the death receptor Fas. In particular, mitochondria-specific negatively charged lipid, 1,3-bis(sn-3′-phosphatidyl)-sn-glycerol (cardiolipin, CL), appeared at the cell surface. Presumably, such changes were connected to the alteration of membrane traffic that is induced early after Fas triggering and occurs independently of the activation of caspases and involves various intracellular organelles including mitochondria (for a review see [15]).

Thus, after death receptor engagement, specific changes in the lipid composition occur not only at the plasma membrane, but also in intracellular membranes. In particular, the most critical changes during apoptosis take place in mitochondria, where they promote the permeabilization of the outer mitochondrial membrane (OMM) to release apoptogenic factors into the cytoplasm [16–18].

Indeed, it is well known that apoptosis is accompanied by mitochondrial perturbations, such as reduction of mitochondrial transmembrane potential and increase of mitochondrial generation of superoxide anion [16–18]. Both events precede nuclear DNA fragmentation. After the apoptotic signal, cells sustain progressive lipid peroxidation, resulting from the generation of lipid-diffusible reactive oxygen species [19]. The major sites of free radical generation include mitochondria, endoplasmic reticulum (ER), and nuclear membranes [19–22]. A structural defect in the inner mitochondrial membrane which incorporates most mature CL has been reported [23, 24]. Two additional mechanisms have been proposed to account for phospholipid movement to mitochondria, which include the involvement of a collision-based mechanism involving the ER and the mitochondria and the transient fusion between ER and mitochondrial membranes [25, 26]. By studying the early dynamics of intracellular membranes in Fas-mediated apoptosis, it has been reported that FasL treatment induces intermixing of Golgi and mitochondrial organelles [27–29]. Fas ligand-stimulated endocytosis also leads to an early and directional “movement” of endocytic vesicles towards the mitochondrial compartment [28]. This scrambling seems to be not an isolated phenomenon, nor restricted to lymphoid cells [27]. The intermixing of membrane organelles also precedes any alteration of the main cytoskeleton components, actin and tubulin. Hence, the scrambling of diverse organelles occurs early after activation of Fas and appears to reflect a global alteration in membrane traffic, being particularly rapid in cells physiologically sensitive to Fas-mediated death. We are thus beginning to understand the early changes in mitochondrial lipids that occur before, or concomitantly with the mitochondrial outer membrane permeabilization [15]. In this* scenario*, membrane lipids, including CL, diacylglycerol (DAG), and lysolipids, play an essential role in facilitating the changes in membrane traffic induced by Fas stimulation in sensitive cells.

However, relatively little is known about the mechanisms and intracellular pathways regulating CL membrane translocation.

## 2. Distribution Changes of Mitochondrial Cardiolipin following Apoptotic Triggering

**Figure 1:**
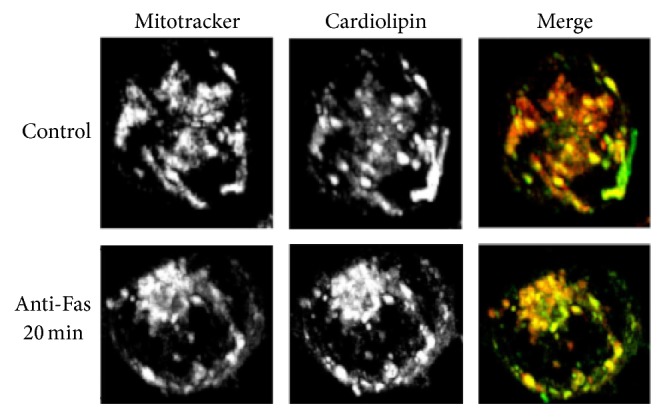
Cardiolipin-mitochondria association following apoptotic triggering. T cells, untreated or treated with anti-CD95/Fas for 20 min, were stained with MCL-BODIPY, a green fluorescent analogue of CL [30, 31], and then with 50 nM Mitotracker red. Projected images from 33 z-sections of 0.2 nm, obtained after 10 cycles of deconvolution, were acquired by using a state-of-the-art Deltavision RT system.

CL is considered to be a specific component of mitochondria, since it is synthesized exclusively within the inner mitochondrial membrane (IMM), where it constitutes about 20% of the total lipid composition [32]. CL is required for optimal mitochondrial function and is known to provide essential structural and functional support to several proteins involved in mitochondrial bioenergetics [30, 32, 33]. Even if CL is present almost exclusively within the IMM, it is also found in the OMM and even more at the contact sites formed between the inner and outer membranes [34]. Redistribution of CL to the OMM became more evident under mild mitochondrial damage, since CL serves as a recognition signal for dysfunctional mitochondria. In particular, it was observed following CD95/Fas triggering. In Figure 1 is reported a typical deconvolution imaging, showing the presence of CL predominantly within mitochondrial membranes in control cells. In cells stimulated with anti-CD95/Fas for 20 min a change in CL distribution was observed, although association with mitochondria remained evident. Changes in CL distribution appear to occur prior or concomitantly to membrane exposure of PS, but after the onset of an overproduction of reactive oxygen species (ROS) [35].

However, peroxidation of CL is far greater in response to severe stress than under normal or mild-damage conditions [36–38]. The accumulation of oxidized CL on the OMM results in recruitment of Bax and formation of the mitochondrial permeability transition pore (MPTP), which releases cytochrome* c* (Cyt* c*) from mitochondria [39]. CL can be considered a versatile phospholipid participating in several mitochondria-dependent apoptotic steps [40], including the modulation of the proapoptotic actions of Bid and other Bcl-2 family proteins [7, 41] through specific interactions [42, 43] (CL and Cyt* c*, t-Bid, and caspase-8) which have now been clarified and the combination of lipid-protein mixtures is becoming evident, that is, raft-like microdomains containing CL, in the expression and regulation of members of the apoptotic machinery [44–49]. In particular, raft-like microdomains may contribute to cell polarization, mitochondrial oxidative phosphorylation, and the release of apoptogenic factors [50–52] by recruitment of Bcl-2 family proteins, including truncated Bid, t-Bid, and Bax following CD95/Fas triggering [50, 51]. Thus, these dynamic structures could act as a sort of signaling device and/or by a “chamber” catalyzing key critical reactions as those determining apoptotic execution pathway [52, 53]. These mitochondrial raft-like microdomains are enriched in gangliosides (GD3) and cholesterol (although with a content lower as compared to plasma membrane), plus some other molecules, such as VDAC-1 and the fission protein hFis1, which are constitutively present. CL may be constitutively present in raft-like microdomains of mitochondria [54], where it acts as a mitochondria-associated platform that is required for caspase-8 translocation, oligomerization, and activation after CD95 stimulation [44]. The observation that CL may be associated with mitochondrial raft-like microdomains is not unexpected considering this lipid has four acyl groups, most of which are highly unsaturated, and two phosphate moieties. Similar findings were obtained by Karbowski et al., who demonstrated a recruitment of Bax to lipid microdomains associated with mitochondrial fission sites during the early steps of staurosporine-induced apoptosis [55].

These data supported the hypothesis that CL is an essential constituent of functional microdomains present within the contact sites between the inner and outer mitochondrial membranes [30, 56–58] from which it may drive the oligomerization and proapoptotic action of death inducing proteins.

## 3. Cardiolipin-Bid Interaction following Apoptotic Triggering

In this respect, CL acts as the mitochondrial receptor for Bid [59], providing specificity for targeting of t-Bid to mitochondria, regulating the oligomerization of Bax [60] and mobilization of cytochrome *c*. It seems plausible that targeting and recruitment of tBid to lipid microdomains, most likely through CL binding, may be necessary for formation of multiprotein complexes which regulate changes in the mitochondrial morphology [43, 47, 61–63].

CL remodelling involves relocation to the outer mitochondrial membrane as well as to extramitochondrial compartments [64], with rapid deacylation into mono- and dilysocardiolipin (with three and two acyl chains, resp.). These metabolites are transported to the endoplasmic reticulum (ER) for efficient reacylation into the mature forms of CL found in mitochondria, in a process that seems to be facilitated by lipid transfer proteins including Bid [43]. Interestingly, Bid has lipid transfer activity between ER and mitochondria, since it preferentially interacts with negatively charged phospholipids like PG [65], which are precursors of CL. This suggests that Bid may be involved in the synthesis or recycling of CL. Indeed, CL biosynthesis has been found to be critically affected in a model of lipid-induced apoptosis [66], consistent with the observation that the mitochondrial content of CL decreases during apoptosis [67].

We can frame the findings that Bid binds to CL [59] and transports its precursors [65] in the context of CL remodelling in mitochondrial membranes, which is likely to be fundamental for their integrity. Preservation or alteration of OMM integrity is essential to the anti- or proapoptotic action of the different proteins of the Bcl-2 family [68]. The first factor that affects these death regulators is their association with the OMM. Some, like Bcl-2 itself, are permanent resident. Conversely, proapoptotic proteins, such as Bid and Bim, predominantly reside in other cell compartments but move to the OMM in response to apoptotic stimuli. Because this mitochondrial relocation is relatively rapid and generally precedes the membrane damage that allows the release of cytochrome *c* into the cytosol, it could derive from some lipid signal generated by upstream activation of phospholipid-mobilising enzymes. So far, emphasis has been put on protein interactions and modifications that can affect the mitochondrial association of proapoptotic Bcl-2 proteins following death signalling [59, 68]. However, several examples exist of proteins that rapidly associate with mitochondria in response to a lipid signal, including Ca^++^-independent phospholipase A_2_ [69] and lipoxygenase [70].

Reversible association with intracellular membranes is typical of proteins that transport lipids, such as fatty acid binding proteins. Interestingly, several fatty acid binding proteins are induced by Bcl-2 overexpression [71]. This evidence has been connected with the antioxidant activity associated with Bcl-2 expression [71], an activity which may be linked to the catabolism of fatty acids [72]. The protective antiperoxidative action of Bcl-2 resembles that of some enzymes, especially phospholipid hydroperoxide glutathione peroxidase, which directly counteracts damaging oxygen radicals [67]. Further highlighting the connection between Bcl-2 proteins and lipids, CL and its remodelling metabolites are very prone to oxidation because they predominantly contain linoleoyl and arachidonoyl fatty acids, which undergo peroxidation processes that are enhanced during apoptosis [67]. However, we and others have found that enhanced peroxidation of CL follows earlier changes in cellular membranes that occur after apoptosis induction by stimulation of death receptors such as Fas, for example [15]. One of these early alterations is CL redistribution at the cell surface after Fas stimulation [64], which may be due to part of the global alteration in membrane traffic that occurs early after death induction. Mass spectrometry has indeed shown that CL and its metabolites relocate from mitochondria to other intracellular organelles during Fas-induced apoptosis. Concomitantly, cytosolic Bid relocated to the light membranes, including the plasma membrane and associated vesicular systems. A direct Bid-CL interaction was demonstrated by the observation that CL and its metabolite monolysoCL coimmunoprecipitated with Bid, especially after Fas stimulation, indicating a dynamic interaction of the protein with CL and its metabolites [64]. The question that has remained unanswered, so far, is whether these changes in intracellular membranes and their lipid components are associated with other processes that are altered early after death receptor stimulation.

## 4. Cardiolipin Association with Cytoskeleton Protein Vimentin following Apoptotic Triggering

Aside from the alteration of intracellular membrane, cell death signalling also induces rearrangement and aggregation of cytoskeletal proteins [73]. These morphological changes could be due to differential reorganization of the fundamental cytoskeletal proteins, actin and tubulin, induced by enzymes activated during cell death, in particular caspases [73]. Since actin-mediated membrane traffic appeared to be the major driver for the scrambling of mitochondrial membranes and their constituent CL, cytoskeleton remodelling has been implicated in apoptosis-induced redistribution of intracellular membrane components [74]. This could be accompanied by the ability of microtubules to have spontaneous changes in the polymerization and depolymerization activities during apoptosis [75]. Interestingly, CL redistribution was associated with the cytoskeleton protein vimentin. Indeed, vimentin and CL can interact on the cell surface of apoptotic cells, thereby forming an immunogenic complex [76]. This binding occurs quite early in the autophagic process and precedes caspase activation, as revealed by kinetics studies on CL exposure on the cell surface [14, 64].

Moreover,* in vitro* studies confirmed that vimentin has high affinity binding with CL. In particular, vimentin was shown to have a stronger interaction with CL compared with other phospholipids, such as phosphatidylcholine and phosphatidylserine [77]. CL-vimentin binding may be attributable to electrostatic interaction between positive charged amino acids of vimentin and negative charged polar head of CL.

Vimentin is a type III cytoskeleton intermediate filament protein that is ubiquitously expressed in mesenchymal cells. The filaments of vimentin interact with elements of the nucleus, endoplasmic reticulum, and mitochondria, playing a very important role in supporting and anchoring various organelles in the cytoplasm. In general, vimentin is considered as a component of the cytoskeleton responsible for the maintenance of cellular integrity [78, 79]. Interestingly, surface-expressed forms of vimentin have been discovered on several cell types, including apoptotic neutrophils and T cells [80, 81], activated macrophages [82], platelets [83], vascular endothelial cells [84], brain microvascular endothelial cells [85], and skeletal muscle cells [76]. In particular, rod and tail domains of vimentin are exposed on the cell surface of human apoptotic T lymphocytes [81], where vimentin anchors to the inner side of plasma membrane by interaction with ankyrin [86]. Boilard et al. [81] also showed that secreted human group IIA phospholipase A_2_ (PLA_2_) binds to vimentin on the cell surface of apoptotic T lymphocytes. The interaction between these two proteins enhanced the activity of PLA_2_, suggesting that vimentin may play a role in PLA_2_-mediated cellular arachidonic acid release [81].

However, the mechanisms by which vimentin reaches the cell surface are not completely known. In this regard, the possible association of vimentin with Bid suggests that this molecule might be involved in the intracellular transport not only of CL and its metabolites, but also of vimentin and potentially account for their relocation onto the plasma membrane of apoptotic cells.

## 5. Cardiolipin Exposure on the Cell Surface during Apoptosis: A Trigger for “Antiphospholipid Antibodies”

Several evidences showed that CL becomes exposed on the plasma membrane of cells undergoing apoptosis induced by death receptors, like CD95/Fas and tumor necrosis factor-alpha (TNF-*α*) [14, 64, 87]. Translocation onto the cell surface implies a leakage of CL (and/or of its metabolites) from the normal remodelling cycle [88], probably as a consequence of an apoptosis-mediated increase of ER and secretory membranes. Interestingly, mass spectroscopy analysis has demonstrated an early degradation of mitochondrial CL into its immediate metabolite, monolysocardiolipin, during CD95/Fas-induced apoptosis [43]. In addition, it revealed that CL and its metabolites relocated from mitochondria to other intracellular organelles during apoptosis, with a conversion into nonmitochondrial lipids. These findings have been subsequently confirmed in human promonocytic U937 cells [64].

Both cytofluorimetric and scanning confocal microscopy analyses revealed that anticardiolipin (aCL) IgG purified from the serum of patients with the Antiphospholipid Antibody Syndrome (APS) binds to CL on the surface of apoptotic cells. This analysis showed that CL molecules are exposed on the cell plasma membrane time-dependently and that their appearance precedes DNA degradation and cell lysis by several hours. This finding suggested that CL molecules may function as self-antigen molecules. Indeed, Casciola-Rosen and coworkers [89] have shown aCL binding to surface blebs of apoptotic cells, which would be consistent with the clustering of aCL immunostaining in focal surface regions that were detected in apoptotic cells. This indicates that cells undergoing apoptosis expose CL on their surface in segregated membrane regions that could enhance the binding of circulating autoantibodies. Since binding of autoantibody to one component of a multicomponent complex can influence the subsequent processing and presentation of the other antigens in the complex [90], it is possible that coating of apoptotic blebs by aCL enhances the immunogenicity of these autoantigens [91]. When apoptosis occurs in a microenvironment in direct contact with the plasma, the procoagulant role of the apoptotic surface may be expressed additionally [92]. Opsonization of apoptotic cells by antiphospholipid antibodies (aPL) has been shown to enhance recognition and phagocytosis by macrophages, with massive TNF-*α* secretion [93, 94]. The release of TNF-*α* may amplify this process by inducing further apoptosis and promoting the maturation of APC towards a more efficient antigen processing and presentation capability.

Thus, during apoptosis, CL becomes exposed on the surface of cells, as revealed by using purified aCL antibodies obtained from patients with APS; this evidence could suggest how CL molecules may function as self-antigen molecules [95].

In fact, aCL antibodies are the hallmark of APS [92] and are used for its diagnosis, but different reports showed that the “true” antigens for aCL binding are phospholipid-binding proteins that are described as phospholipid cofactors [96, 97]. However, the chemistry of the acyl chains of CL is important not only for the intrinsic immunogenicity of CL molecule, but also for the binding to the phospholipid cofactors, including *β*2-glycoprotein I (*β*2-GPI) [98, 99].

Vimentin, similarly to *β*2-GPI, could therefore act as a cofactor for the presentation of CL to the immune system, potentially enhancing its antigenicity. Recently, a proteomic approach identified vimentin as the main endothelial molecule recognized by aPL [77]. Interestingly, almost all the APS patients displayed the presence of anti-vimentin/CL complex antibodies. This finding suggests that vimentin may be considered a “new” antigenic cofactor for aPL in APS. This finding is not completely surprising because a significant correlation between anti-vimentin and aCL antibodies has been already reported [100]. Moreover, their particular role in the pathogenesis of thrombotic events in autoimmune diseases has been described [31]. In particular, Leong et al. demonstrated that anti-vimentin antibodies lead to activation of platelets and leukocytes, as revealed by induced expression of P-selectin, fibrinogen, tissue factor, and formation of platelet-leukocyte conjugates via platelet-activating factor [101]. Furthermore, platelet vimentin may regulate fibrinolysis in plasma and thrombus formation by binding platelet-derived fibronectin-plasminogen activator inhibitor complexes [102]. Moreover, anti-vimentin/CL antibodies may exert their pathogenic role by triggering a signal transduction pathway involving Toll-like receptor 4, IRAK phosphorylation, and NF-*κ*B activation, with consequent release of proinflammatory and procoagulant factors [77].

**Figure 2:**
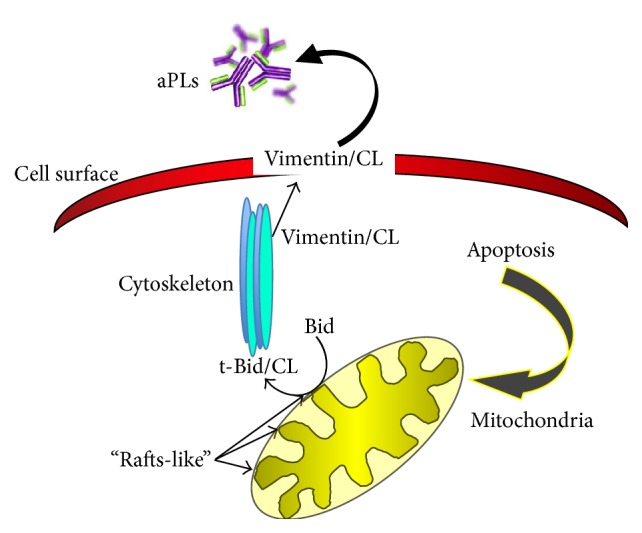
Schematic drawing depicting the intracellular traffic of cardiolipin and its metabolites following apoptotic triggering.

In any case, the mechanism through which the vimentin/CL acquires an antigenic power is still unknown. Despite this, apoptosis is a pathophysiological mechanism which determines the exposure to the plasma membrane not only of CL, but also of vimentin (Figure 2) [14, 80].

The presence of autoantibodies in some systemic diseases may arise from abnormal exposure of autoantigens on apoptotic cells. In fact, in many autoimmune diseases, such as Systemic Lupus Erythematosus (SLE) or APS, it is possible to detect the presence of high amounts of apoptotic cells compared to the control samples [103]. In this regard, apoptotic cells may provide an abundant source of antigens, and their exposure on the cell surface of apoptotic cells may represent an* in vivo* trigger for the production of autoantibodies.

## 6. Conclusions

Translocation of CL on the surface of apoptotic cells and its interaction with protein cofactor(s) such as vimentin produce a new twist in the ever evolving APS field, because it represents a novel potential trigger for “antiphospholipid antibodies.” Vimentin/CL complex as a “new” target antigen in APS unveils possible lines of therapeutic intervention in those patients with clinical signs suggestive of APS, who are persistently negative for the conventional tests but positive for anti-vimentin/CL antibodies [77, 104]. In addition, the knowledge of new antigenic targets may contribute to pointing out the risk stratification of the disease, taking into account first the potential combinations/panels of available aPL tests.

## References

[1] Kerr J. F., Wyllie A. H., Currie A. R. (1972). Apoptosis: a basic biological phenomenon with wide-ranging implications in tissue kinetics. *British Journal of Cancer*.

[2] Häcker G. (2000). The morphology of apoptosis. *Cell and Tissue Research*.

[3] Hengartner M. O. (2000). The biochemistry of apoptosis. *Nature*.

[4] Boya P., Andreau K., Poncet D. (2003). Lysosomal membrane permeabilization induces cell death in a mitochondrion-dependent fashion. *The Journal of Experimental Medicine*.

[5] Peitsch M. C., Mannherz H. G., Tschopp J. (1994). The apoptosis endonucleases: cleaning up after cell death?. *Trends in Cell Biology*.

[6] Taylor R. C., Cullen S. P., Martin S. J. (2008). Apoptosis: controlled demolition at the cellular level. *Nature Reviews Molecular Cell Biology*.

[7] Cristea I. M., Esposti M. D. (2004). Membrane lipids and cell death: an overview. *Chemistry and Physics of Lipids*.

[8] Yamaji-Hasegawa A., Tsujimoto M. (2006). Asymmetric distribution of phospholipids in biomembranes. *Biological and Pharmaceutical Bulletin*.

[9] Fadok V. A., Voelker D. R., Campbell P. A., Cohen J. J., Bratton D. L., Henson P. M. (1992). Exposure of phosphatidylserine on the surface of apoptotic lymphocytes triggers specific recognition and removal by macrophages. *The Journal of Immunology*.

[10] Martin S. J., Reutelingsperger C. P. M., McGahon A. J. (1995). Early redistribution of plasma membrane phosphatidylserine is a general feature of apoptosis regardless of the initiating stimulus: inhibition by overexpression of Bcl-2 and Abl. *Journal of Experimental Medicine*.

[11] Fadok V. A., Bratton D. L., Rose D. M., Pearson A., Ezekewitz R. A. B., Henson P. M. (2000). A receptor for phosphatidylserine-specific clearance of apoptotic cells. *Nature*.

[12] Mitchell J. E., Cvetanovic M., Tibrewal N. (2006). The presumptive phosphatidylserine receptor is dispensable for innate anti-inflammatory recognition and clearance of apoptotic cells. *The Journal of Biological Chemistry*.

[13] Liu J., Epand R. F., Durrant D. (2008). Role of phospholipid scramblase 3 in the regulation of tumor necrosis factor-alpha-induced apoptosis. *Biochemistry*.

[14] Sorice M., Circella A., Misasi R. (2000). Cardiolipin on the surface of apoptotic cells as a possible trigger for antiphospholipid antibodies. *Clinical and Experimental Immunology*.

[15] Crimi M., Esposti M. D. (2011). Apoptosis-induced changes in mitochondrial lipids. *Biochimica et Biophysica Acta*.

[16] Zamzami N., Susin S. A., Marchetti P. (1996). Mitochondrial control of nuclear apoptosis. *The Journal of Experimental Medicine*.

[17] Vayssière J.-L., Petit P. X., Risler Y., Mignotte B. (1994). Commitment to apoptosis is associated with changes in mitochondrial biogenesis and activity in cell lines conditionally immortalized with simian virus 40. *Proceedings of the National Academy of Sciences of the United States of America*.

[18] Petit P. X., Lecoeur H., Zorn E., Dauguet C., Mignotte B., Gougeon M.-L. (1995). Alterations in mitochondrial structure and function are early events of dexamethasone-induced thymocyte apoptosis. *Journal of Cell Biology*.

[19] Hockenbery D. M., Oltvai Z. N., Yin X.-M., Milliman C. L., Korsmeyer S. J. (1993). Bcl-2 functions in an antioxidant pathway to prevent apoptosis. *Cell*.

[20] Boveris A., Chance B. (1973). The mitochondrial generation of hydrogen peroxide. General properties and effect of hyperbaric oxygen. *Biochemical Journal*.

[21] Casciola-Rosen L. A., Miller D. K., Anhalt G. J., Rosen A. (1994). Specific cleavage of the 70-kDa protein component of the U1 small nuclear ribonucleoprotein is a characteristic biochemical feature of apoptotic cell death. *The Journal of Biological Chemistry*.

[22] Casiano C. A., Martin S. J., Green D. R., Tan E. M. (1996). Selective cleavage of nuclear autoantigens during CD95 (Fas/APO-1)- mediated T cell apoptosis. *The Journal of Experimental Medicine*.

[23] Macho A., Castedo M., Marchetti P. (1995). Mitochondrial dysfunctions in circulating T lymphocytes from human immunodeficiency virus-1 carriers. *Blood*.

[24] Schlame M., Rua D., Greenberg M. L. (2000). The biosynthesis and functional role of cardiolipin. *Progress in Lipid Research*.

[25] Moreau P., Cassagne C. (1994). Phospholipid trafficking and membrane biogenesis. *Biochimica et Biophysica Acta*.

[26] Zachowski A. (1993). Phospholipids in animal eukaryotic membranes: transverse asymmetry and movement. *Biochemical Journal*.

[27] Ouasti S., Matarrese P., Paddon R. (2007). Death receptor ligation triggers membrane scrambling between Golgi and mitochondria. *Cell Death and Differentiation*.

[28] Matarrese P., Manganelli V., Garofalo T. (2008). Endosomal compartment contributes to the propagation of CD95/Fas-mediated signals in type II cells. *Biochemical Journal*.

[29] Degli Esposti M. (2008). Organelle intermixing and membrane scrambling in cell death. *Methods in Enzymology*.

[30] Robinson N. C. (1993). Functional binding of cardiolipin to cytochrome c oxidase. *Journal of Bioenergetics and Biomembranes*.

[31] Mahesh B., Leong H.-S., McCormack A., Sarathchandra P., Holder A., Rose M. L. (2007). Autoantibodies to vimentin cause accelerated rejection of cardiac allografts. *The American Journal of Pathology*.

[32] Houtkooper R. H., Vaz F. M. (2008). Cardiolipin, the heart of mitochondrial metabolism. *Cellular and Molecular Life Sciences*.

[33] Mileykovskaya E., Zhang M., Dowhan W. (2005). Cardiolipin in energy transducing membranes. *Biochemistry*.

[34] Ardail D., Privat J.-P., Egret-Charlier M., Levrat C., Lerme F., Louisot P. (1990). Mitochondrial contact sites: lipid composition and dynamics. *The Journal of Biological Chemistry*.

[35] Fernandez M. G., Troiano L., Moretti L. (2002). Early changes in intramitochondrial cardiolipin distribution during apoptosis. *Cell Growth & Differentiation*.

[36] Chicco A. J., Sparagna G. C. (2007). Role of cardiolipin alterations in mitochondrial dysfunction and disease. *The American Journal of Physiology—Cell Physiology*.

[37] Vreken P., Valianpour F., Nijtmans L. G. (2000). Defective remodeling of cardiolipin and phosphatidylglycerol in Barth syndrome. *Biochemical and Biophysical Research Communications*.

[38] Fariss M. W., Chan C. B., Patel M., Van Houten B., Orrenius S. (2005). Role of mitochondria in toxic oxidative stress. *Molecular Interventions*.

[39] Li X. X., Tsoi B., Li Y. F., Kurihara H., He R. R. (2015). Cardiolipin and its different properties in mitophagy and apoptosis. *Journal of Histochemistry & Cytochemistry*.

[40] Gonzalvez F., Gottlieb E. (2007). Cardiolipin: setting the beat of apoptosis. *Apoptosis*.

[41] Kuwana T., Mackey M. R., Perkins G. (2002). Bid, Bax, and lipids cooperate to form supramolecular openings in the outer mitochondrial membrane. *Cell*.

[42] Roucou X., Montessuit S., Antonsson B., Martinou J.-C. (2002). Bax oligomerization in mitochondrial membranes requires tBid (caspase-8-cleaved Bid) and a mitochondrial protein. *Biochemical Journal*.

[43] Degli Esposti M., Cristea I. M., Gaskell S. J., Nakao Y., Dive C. (2003). Proapoptotic Bid binds to monolysocardiolipin, a new molecular connection between mitochondrial membranes and cell death. *Cell Death & Differentiation*.

[44] Gonzalvez F., Schug Z. T., Houtkooper R. H. (2008). Cardiolipin provides an essential activating platform for caspase-8 on mitochondria. *Journal of Cell Biology*.

[45] Jalmar O., García-Sáez A. J., Berland L., Gonzalvez F., Petit P. X. (2010). Giant unilamellar vesicles (GUVs) as a new tool for analysis of caspase-8/Bid-FL complex binding to cardiolipin and its functional activity. *Cell Death and Disease*.

[46] Jalmar O., François-Moutal L., García-Sáez A.-J. (2013). Caspase-8 binding to cardiolipin in giant unilamellar vesicles provides a functional docking platform for Bid. *PLoS ONE*.

[47] Kantari C., Walczak H. (2011). Caspase-8 and Bid: caught in the act between death receptors and mitochondria. *Biochimica et Biophysica Acta*.

[48] Schug Z. T., Gottlieb E. (2009). Cardiolipin acts as a mitochondrial signalling platform to launch apoptosis. *Biochimica et Biophysica Acta*.

[49] Oh K. J., Barbuto S., Meyer N., Kim R.-S., Collier R. J., Korsmeyer S. J. (2005). Conformational changes in BID, a pro-apoptotic BCL-2 family member, upon membrane binding. A site-directed spin labeling study. *The Journal of Biological Chemistry*.

[50] Garofalo T., Giammarioli A. M., Misasi R. (2005). Lipid microdomains contribute to apoptosis-associated modifications of mitochondria in T cells. *Cell Death & Differentiation*.

[51] Malorni W., Garofalo T., Tinari A. (2008). Analyzing lipid raft dynamics during cell apoptosis. *Methods in Enzymology*.

[52] Malorni W., Giammarioli A. M., Garofalo T., Sorice M. (2007). Dynamics of lipid raft components during lymphocyte apoptosis: the paradigmatic role of GD3. *Apoptosis*.

[53] Garofalo T., Tinari A., Matarrese P. (2007). Do mitochondria act as ‘cargo boats’ in the journey of GD3 to the nucleus during apoptosis?. *FEBS Letters*.

[54] Sorice M., Manganelli V., Matarrese P. (2009). Cardiolipin-enriched raft-like microdomains are essential activating platforms for apoptotic signals on mitochondria. *FEBS Letters*.

[55] Karbowski M., Lee Y.-J., Gaume B. (2002). Spatial and temporal association of Bax with mitochondrial fission sites, Drp1, and Mfn2 during apoptosis. *Journal of Cell Biology*.

[56] Hovius R., Thijssen J., van der Linden P., Nicolay K., de Kruijff B. (1993). Phospholipid asymmetry of the outer membrane of rat liver mitochondria. Evidence for the presence of cardiolipin on the outside of the outer membrane. *FEBS Letters*.

[57] Nicolay K., Rojo M., Wallimann T., Demel R., Hovius R. (1990). The role of contact sites between inner and outer mitochondrial membrane in energy transfer. *Biochimica et Biophysica Acta*.

[58] Hovius R., Lambrechts H., Nicolay K., de Kruijff B. (1990). Improved methods to isolate and subfractionate rat liver mitochondria. Lipid composition of the inner and outer membrane. *Biochimica et Biophysica Acta*.

[59] Lutter M., Fang M., Luo X., Nishijima M., Xie X.-S., Wang X. (2000). Cardiolipin provides specificity for targeting of tBid to mitochondria. *Nature Cell Biology*.

[60] Rostovtseva T. K., Boukari H., Antignani A. (2009). Bax activates endophilin B1 oligomerization and lipid membrane vesiculation. *The Journal of Biological Chemistry*.

[61] Manara A., Lindsay J., Marchioretto M., Manara A. (2009). Bid binding to negatively charged phospholipids may not be required for its pro-apoptotic activity in vivo. *Biochimica et Biophysica Acta*.

[62] Kudla G., Montessuit S., Eskes R. (2000). The destabilization of lipid membranes induced by the C-terminal fragment of caspase 8-cleaved Bid is inhibited by the N-terminal fragment. *The Journal of Biological Chemistry*.

[63] Grinberg M., Sarig R., Zaltsman Y. (2002). tBID homooligomerizes in the mitochondrial membrane to induce apoptosis. *The Journal of Biological Chemistry*.

[64] Sorice M., Circella A., Cristea I. M. (2004). Cardiolipin and its metabolites move from mitochondria to other cellular membranes during death receptor-mediated apoptosis. *Cell Death and Differentiation*.

[65] Esposti M. D., Erler J. T., Hickman J. A., Dive C. (2001). Bid, a widely expressed proapoptotic protein of the Bcl-2 family, displays lipid transfer activity. *Molecular and Cellular Biology*.

[66] Ostrander D. B., Sparagna G. C., Amoscato A. A., McMillin J. B., Dowhan W. (2001). Decreased cardiolipin synthesis corresponds with cytochrome c release in palmitate-induced cardiomyocyte apoptosis. *The Journal of Biological Chemistry*.

[67] Nomura K., Imai H., Koumura T., Kobayashi T., Nakagawa Y. (2000). Mitochondrial phospholipid hydroperoxide glutathione peroxidase inhibits the release of cytochrome c from mitochondria by suppressing the peroxidation of cardiolipin in hypoglycaemia-induced apoptosis. *Biochemical Journal*.

[68] Martinou J.-C., Green D. R. (2001). Breaking the mitochondrial barrier. *Nature Reviews Molecular Cell Biology*.

[69] Atsumi G.-I., Tajima M., Hadano A., Nakatani Y., Murakami M., Kudot I. (1998). Fas-induced arachidonic acid release is mediated by Ca^2+^-independent phospholipase A_2_ but not cytosolic phospholipase A_2_, which undergoes proteolytic inactivation. *The Journal of Biological Chemistry*.

[70] Maccarrone M., Melino G., Finazzi-Agrò A. (2001). Lipoxygenases and their involvement in programmed cell death. *Cell Death & Differentiation*.

[71] Voehringer D. W., Hirschberg D. L., Xiao J. (2000). Gene microarray identification of redox and mitochondrial elements that control resistance or sensitivity to apoptosis. *Proceedings of the National Academy of Sciences of the United States of America*.

[72] Esposti M. D., Hatzinisiriou I., McLennan H., Ralph S. (1999). Bcl-2 and mitochondrial oxygen radicals. New approaches with reactive oxygen species-sensitive probes. *The Journal of Biological Chemistry*.

[73] Huot J., Houle F., Rousseau S., Deschesnes R. G., Shah G. M., Landry J. (1998). SAPK2/p38-dependent F-actin reorganization regulates early membrane blebbing during stress-induced apoptosis. *Journal of Cell Biology*.

[74] Esposti M. D., Tour J., Ouasti S. (2009). Fas death receptor enhances endocytic membrane traffic converging into the golgi region. *Molecular Biology of the Cell*.

[75] Blagosklonny M. V., Giannakakou P., El-Deiry W. S. (1997). Raf-1/bcl-2 phosphorylation: a step from microtubule damage to cell death. *Cancer Research*.

[76] Thébault S., Gilbert D., Hubert M. (2002). Orderly pattern of development of the autoantibody response in (New Zealand White × BXSB)F_1_ lupus mice: characterization of target antigens and antigen spreading by two-dimensional gel electrophoresis and mass spectrometry. *The Journal of Immunology*.

[77] Ortona E., Capozzi A., Colasanti T. (2010). Vimentin/cardiolipin complex as a new antigenic target of the antiphospholipid syndrome. *Blood*.

[78] Katsumoto T., Mitsushima A., Kurimura T. (1990). The role of the vimentin intermediate filaments in rat 3Y1 cells elucidated by immunoelectron microscopy and computer-graphic reconstruction. *Biology of the Cell*.

[79] Goldman R. D., Khuon S., Chou Y. H., Opal P., Steinert P. M. (1996). The function of intermediate filaments in cell shape and cytoskeletal integrity. *Journal of Cell Biology*.

[80] Moisan E., Girard D. (2006). Cell surface expression of intermediate filament proteins vimentin and lamin B1 in human neutrophil spontaneous apoptosis. *Journal of Leukocyte Biology*.

[81] Boilard E., Bourgoin S. G., Bernatchez C., Surette M. E. (2003). Identification of an autoantigen on the surface of apoptotic human T cells as a new protein interacting with inflammatory group IIA phospholipase A_2_. *Blood*.

[82] Mor-Vaknin N., Punturieri A., Sitwala K., Markovitz D. M. (2003). Vimentin is secreted by activated macrophages. *Nature Cell Biology*.

[83] Podor T. J., Singh D., Chindemi P. (2002). Vimentin exposed on activated platelets and platelet microparticles localizes vitronectin and plasminogen activator inhibitor complexes on their surface. *The Journal of Biological Chemistry*.

[84] Xu B., deWaal R. M., Mor-Vaknin N., Hibbard C., Markovitz D. M., Kahn M. L. (2004). The endothelial cell-specific antibody PAL-E identifies a secreted form of vimentin in the blood vasculature. *Molecular and Cellular Biology*.

[85] Bryant A. E., Bayer C. R., Huntington J. D., Stevens D. L. (2006). Group A streptococcal myonecrosis: increased vimentin expression after skeletal-muscle injury mediates the binding of *Streptococcus pyogenes*. *The Journal of Infectious Diseases*.

[86] Traub P., Perides G., Schimmel H., Scherbarth A. (1986). Interaction *in vitro* of nonepithelial intermediate filament proteins with total cellular lipids, individual phospholipids, and a phospholipid mixture. *The Journal of Biological Chemistry*.

[87] Alessandri C., Sorice M., Bombardieri M. (2006). Antiphospholipid reactivity against cardiolipin metabolites occurring during endothelial cell apoptosis. *Arthritis Research and Therapy*.

[88] Degli Esposti M. (2002). Lipids, cardiolipin and apoptosis: a greasy licence to kill. *Cell Death and Differentiation*.

[89] Casciola-Rosen L. A., Anhalt G., Rosen A. (1994). Autoantigens targeted in systemic lupus erythematosus are clustered in two populations of surface structures on apoptotic keratinocytes. *Journal of Experimental Medicine*.

[90] Lanzavecchia A. (1995). How can cryptic epitopes trigger autoimmunity?. *Journal of Experimental Medicine*.

[91] Casciola-Rosen L., Rosen A., Petri M., Schlissel M. (1996). Surface blebs on apoptotic cells are sites of enhanced procoagulant activity: implications for coagulation events and antigenic spread in systemic lupus erythematosus. *Proceedings of the National Academy of Sciences of the United States of America*.

[92] Hughes G. R. V. (1985). The anticardiolipin syndrome. *Clinical and Experimental Rheumatology*.

[93] Manfredi A. A., Rovere P., Galati G. (1998). Apoptotic cell clearance in systemic lupus erythematosus. I. Opsonization by antiphospholipid antibodies. *Arthritis and Rheumatism*.

[94] Manfredi A. A., Rovere P., Heltai S. (1998). Apoptotic cell clearance in systemic lupus erythematosus. II. Role of *β*2-glycoprotein I. *Arthritis & Rheumatism*.

[95] Sorice M., Griggi T., Circella A. (1994). Detection of antiphospholipid antibodies by immunostaining on thin layer chromatography plates. *Journal of Immunological Methods*.

[96] Lim W. (2013). Antiphospholipid syndrome. *Hematology*.

[97] Favaloro E. J., Wong R. C. (2014). Antiphospholipid antibody testing for the antiphospholipid syndrome. *Pathology*.

[98] McNeil H. P., Simpson R. J., Chesterman C. N., Krilis S. A. (1990). Anti-phospholipid antibodies are directed against a complex antigen that includes a lipid-binding inhibitor of coagulation: *β*2-Glycoprotein I (apolipoprotein H). *Proceedings of the National Academy of Sciences of the United States of America*.

[99] Matsuura E., Igarashi Y., Fujimoto M., Ichikawa K., Koike T. (1990). Anticardiolipin cofactor(s) and differential diagnosis of autoimmune disease. *The Lancet*.

[100] Blaschek M. A., Boehme M., Jouquan J. (1988). Relation of antivimentin antibodies to anticardiolipin antibodies in systemic lupus erythematosus. *Annals of the Rheumatic Diseases*.

[101] Leong H. S., Mahesh B. M., Day J. R. (2008). Vimentin autoantibodies induce platelet activation and formation of platelet-leukocyte conjugates via platelet-activating factor. *Journal of Leukocyte Biology*.

[102] Podor T. J., Singh D., Chindemi P. (2002). Vimentin exposed on activated platelets and platelet microparticles localizes vitronectin and plasminogen activator inhibitor complexes on their surface. *Journal of Biological Chemistry*.

[103] Emlen W., Niebur J., Kadera R. (1994). Accelerated *in vitro* apoptosis of lymphocytes from patients with systemic lupus erythematosus. *The Journal of Immunology*.

[104] Conti F., Capozzi A., Truglia S. (2014). The mosaic of ‘seronegative’ antiphospholipid syndrome. *Journal of Immunology Research*.

